# The Structural Characteristics and Bioactivity Stability of *Cucumaria frondosa* Intestines and Ovum Hydrolysates Obtained by Different Proteases

**DOI:** 10.3390/md21070395

**Published:** 2023-07-06

**Authors:** Qiuting Wang, Gongming Wang, Chuyi Liu, Zuli Sun, Ruimin Li, Jiarun Gao, Mingbo Li, Leilei Sun

**Affiliations:** 1 College of Life Science, Yantai University, Yantai 264005, China; wqt1981979471@163.com (Q.W.); 17861135192@163.com (R.L.); 15863805335@163.com (J.G.); sllshd1991@163.com (M.L.); 2 Yantai Key Laboratory of Quality and Safety Control and Deep Processing of Marine Food, Shandong Marine Resource and Environment Research Institute, Yantai 264006, China; wgmsd105@163.com; 3 Marine Biomedical Research Institute of Qingdao, Qingdao 266073, China; liucy@ouc.edu.cn; 4 College of Health, Yantai Nanshan University, Yantai 265713, China; sunzuli98@163.com

**Keywords:** *Cucumaria frondosa* intestines, ovum hydrolysate, protease, structural characteristic, bioactivity, simulated gastrointestinal digestion

## Abstract

The study aimed to investigate the effects of alcalase, papain, flavourzyme, and neutrase on the structural characteristics and bioactivity stability of *Cucumaria frondosa* intestines and ovum hydrolysates (CFHs). The findings revealed that flavourzyme exhibited the highest hydrolysis rate (51.88% ± 1.87%). At pH 2.0, the solubility of hydrolysate was the lowest across all treatments, while the solubility at other pH levels was over 60%. The primary structures of hydrolysates of different proteases were similar, whereas the surface hydrophobicity of hydrolysates was influenced by the types of proteases used. The hydrolysates produced by different proteases were also analyzed for their absorption peaks and antioxidant activity. The hydrolysates of flavourzyme had β-fold absorption peaks (1637 cm^−1^), while the neutrase and papain hydrolysates had N-H bending vibrations. The tertiary structure of CFHs was unfolded by different proteases, exposing the aromatic amino acids and red-shifting of the λ-peak of the hydrolysate. The alcalase hydrolysates showed better antioxidant activity in vitro and better surface hydrophobicity than the other hydrolysates. The flavourzyme hydrolysates displayed excellent antioxidant stability and pancreatic lipase inhibitory activity during gastrointestinal digestion, indicating their potential use as antioxidants in the food and pharmaceutical industries.

## 1. Introduction

Sea cucumbers, a member of the phylum Echinodermata, are widely distributed in both tropical and temperate waters, ranging from intertidal zones to the colder depths of the ocean [1]. Sea cucumbers are known for their abundance of nutrients, including sea cucumber peptides, polysaccharides, saponins, vitamins, and trace elements [2]. Research has shown that these compounds have multiple activities, having antioxidant [3], anti-bacterial [4], anticancer, antitumor, hypoglycemic [5], hypolipidemic [6], and hypotensive effects [7]. The *Cucumaria frondosa*, also known as the Icelandic red cucumber or North Atlantic sea cucumber, is a spiny-skinned marine animal belonging to the sea cucumber family Cucumariidae. The species is the most abundant and widely distributed on the east coast of Canada [8]. *Cucumaria frondosa* is primarily grown in Iceland near the Arctic Circle. It grows at a depth of approximately 30 feet in the North Atlantic Ocean, with surface water temperatures not exceeding 4 °C. The *Cucumaria frondosa* generally reaches over 10 years of age [9]. Cucumaria frondosa is a sea cucumber variety that boasts superior quality due to its minimal pollutant content and rich nutrient accumulation [10]. 

However, after harvest, *Cucumaria frondosa* is commonly gutted, which involves removing all internal organs, including the respiratory tract, ovum, and intestines, accounting for approximately 50% of the total weight. Tripoteau et al. [11] demonstrated the in vitro antiviral activity of *Cucumaria frondosa*, while Senadheera et al. [9] demonstrated the antioxidant activity of hydrolyzed proteins from the body parts of the North Atlantic sea cucumber. Unfortunately, despite the presence of various bioactive compounds, the offal of *Cucumaria frondosa* is discarded entirely as waste, and the byproducts of *Cucumaria frondosa* are under-exploited in comparison to other echinoderm species, resulting in significant waste [12]. 

Proteins can serve as a functional substance, but studies have demonstrated that the enzymatic hydrolysates of proteins exhibit higher biological activity [13]. The physicochemical properties and biological activity of hydrolysates depend mainly on the types of proteases and the hydrolysis process [14]. However, limited research has been conducted on the effects of different enzymatic hydrolyses on the structure, physicochemical properties, and bioactivity of *Cucumaria frondosa* intestines and ovum.

The study aims to investigate the impact of different proteases on the structural characteristics and biological activities of hydrolysates. Four hydrolysates, prepared using alcalase, papain, flavourzyme, and neutrase, were selected based on their degree of hydrolysis. The hydrolysates were evaluated for their antioxidant potential and pancreatic lipase inhibitory activity. Additionally, the stability of their bioactivity was assessed after in vitro gastrointestinal digestion. The findings of this study determine the optimal protease for producing hydrolysates from the byproducts of *Cucumaria frondosa* and provide theoretical support for their industrial use.

## 2. Results and Discussion

### 2.1. Degree of Hydrolysis (DH) of Hydrolysates Obtained by Different Proteases

**Figure 1:**
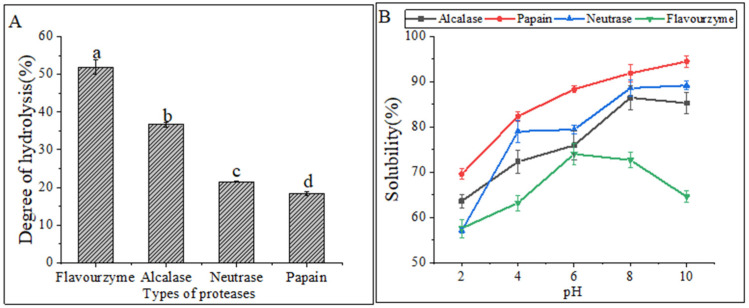
(**A**) Degrees of hydrolysis of different proteases. The lowercase letters indicate significant differences (*p* < 0.05) in the DHs of CFHs obtained by different proteases’ hydrolysis. (**B**) Solubility of hydrolysates.

Figure 1A displayed the DH values of four proteases under their respective optimal conditions. The highest DH was observed in flavourzyme hydrolysates (51.88% ± 1.86%), followed by alcalase hydrolysates (36.61% ± 0.60%) and neutrase hydrolysates (21.43% ± 0.14%), while papain hydrolysates (18.33% ± 0.46%) had the lowest DH. The DH values were found to be significantly different (*p* < 0.05) among the four CFHs. Alcalase, a deep endopeptidase, primarily cleaves peptide bonds at the C-terminal polypeptide bond of hydrophobic amino acids. The DH of the hydrolysates varied depending on the types of proteases used. The DHs of the flavourzyme and alcalase hydrolysates were found to be higher compared to those of neutrase and papain. The difference can be attributed to the fact that alcalase acts as an endopeptidase with a serine active site, whereas flavourzyme is both an exopeptidase and endopeptidase of a cysteine protease with a leucine aminopeptidase [15].

### 2.2. Solubility of Hydrolysates Obtained by Different Proteases

Hydrolysates′ solubility is a crucial physicochemical property that significantly influences their functional properties [16]. The solubility of hydrolysates prepared at various pH levels (2.0–10.0) is shown in Figure 1B. The solubility of the hydrolysates was the lowest for all treatments at a pH of 2.0. Enzymatic digestion alters the hydrophobicity of protein hydrolysates by affecting the balance of the hydrophilic and hydrophobic groups of the hydrolysates, as well as the release of polar and ionized groups [17]. In general, the solubility improved as the pH shifted toward basic conditions. Similar solubility profiles were observed in protein hydrolysates prepared from body wall of the North Atlantic sea cucumber [9].

### 2.3. Structural Characteristics of Hydrolysates

#### 2.3.1. Surface Hydrophobicity

**Figure 2:**
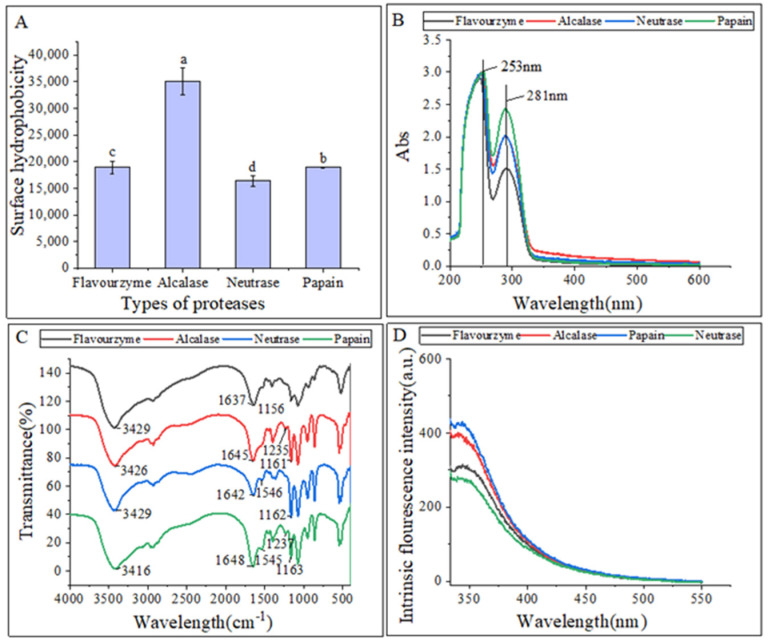
(**A**) Surface hydrophobicity of hydrolysates; (**B**) UV–vis spectra of hydrolysates; (**C**) FTIR spectra of hydrolysates; (**D**) intrinsic fluorescence spectroscopy of hydrolysates. The lowercase letters indicate significant differences (*p* < 0.05) in the surface hydrophobicity of CFHs obtained by different proteases’ hydrolysis.

The surface hydrophobicity of the hydrolysates is shown in Figure 2A. Gbemisola et al. [18] found that the surface hydrophobicity of a protein depended on its spatial conformations and the amount of amino acids exposed during proteolysis. Peptidases can ruin hydrophobic areas, making them more hydrophilic and thus improving the dispersibility of the hydrolysates in water. The alcalase hydrolysates had a significantly higher surface hydrophobicity compared to the other three proteases, indicating a higher concentration of aromatic amino acids. This finding was consistent with that of Zohreh et al. [13]. 

#### 2.3.2. Ultraviolet-Visible (UV-Vis) Spectroscopy Analysis

Figure 2B displayed that the UV-Vis spectra of these hydrolysates were quite similar, with strong absorption peaks at 260 nm and 280 nm. This could be attributed to the influence of tyrosine (278 nm) and phenylalanine (257 nm). The hydrolysates exhibited a strong absorption peak around 280 nm, which is characteristic of hydrophobic amino acids such as tyrosine, phenylalanine, and tryptophan, indicating the hydrophobic property of the extracted hydrolysates [19].

#### 2.3.3. Fourier Transform Infrared Spectroscopy (FTIR) Analysis

The differences in the secondary structures of the hydrolysates were analyzed using FTIR. The FTIR spectra of hydrolysates from different proteases are presented in Figure 2C. FTIR is commonly used to examine peptides and proteins since it can detect the amide (peptide) bonds, which exhibit distinct IR signals for folded peptides and proteins [20]. The vibrational frequency is determined by the hydrogen bonding nature between C=O and C-N in the amide-I band (1600–1700 cm^−1^). The α-helix structure is identified by the absorption peak at 1650–1658 cm^−1^ in the amide-I band, while the β-fold structure is identified by the peak at 1610–1640 cm^−1^. The random curl structure is identified by the peak at 1640–1650 cm^−1^, and the β-turn structure is identified by the peak at 1660–1695 cm^−1^. In the amide III region, the β-fold structure is identified by the peak at 1181–1248 cm^−1^, the β-turn structure is identified by the peak at 1270–1295 cm^−1^, and the irregular curl structure is identified by the peak at 1255–1288 cm^−1^. The C=O stretching vibration absorption peak is located at 1630–1680 cm^−1^ in the peptide bond, while the N-H bending vibration peak is located around 1550 cm^−1^. The N-H stretching vibrations are identified by the absorption peak around 3100–3500 cm^−1^ [21]. 

The FTIR spectra analysis revealed that hydrolysates treated with different proteases exhibited changes in their spectra, with only slight shifts in bands. Specifically, hydrolysates treated with alcalase, neutrase, and papain displayed irregular curls at 1645 cm^−1^, 1642 cm^−1^, and 1648 cm^−1^, respectively, whereas hydrolysates treated with flavourzyme exhibited mainly β-fold absorption peaks at 1637 cm^−1^. The α-helix structure, an ordered structure, is easily influenced by conformational changes. On the other hand, the β-sheet and β-turn structures are also ordered structures but with relative stretches, whereas the random coil structure is a disordered structure. The decrease in β-sheet structures in the hydrolysates and the increase in random coil structures indicated that the protease treatments caused the ordered structure of CFHs to become disordered. Similarly, the structure of mung bean protein enzymatic hydrolysates was analyzed, and it was found that the neutrase and papain hydrolysates showed N-H bending vibrations, while the flavourzyme and alcalase hydrolysates did not, suggesting that the N-H bending vibrations were disrupted during the enzymatic digestion of the flavourzyme and alcalase [22].

#### 2.3.4. Intrinsic Fluorescence Spectroscopy Analysis

Intrinsic fluorescence spectroscopy is a sensitive technique used to detect conformational changes in the tertiary structure of proteins [23]. This is achieved by exciting the aromatic amino acid residues (Trp, Tyr, and Phe) with excitation light, which produces fluorescence [24]. Figure 2D shows that the fluorescence emission spectra of flavourzyme, alcalase, and neutrase hydrolysates were red-shifted as compared to those of papain. This shift might be attributed to changes in the protein structure after enzymatic hydrolysis. The side-chain groups of the aromatic amino acid residues that were originally buried in the protein were gradually exposed to the molecular surface, resulting in a change in the polar environment of the tryptophan residues and leading to the red shift of the peak.

### 2.4. Antioxidant Activity of Hydrolysates Obtained by Different Proteases

**Figure 3:**
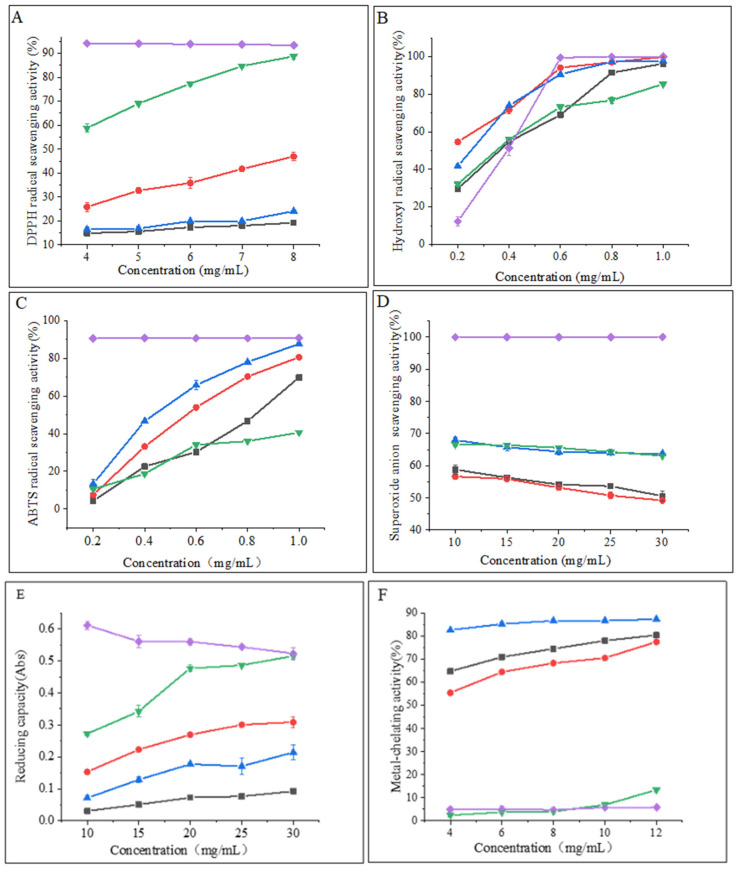
(**A**) DPPH radical scavenging activity of hydrolysates; (**B**) hydroxyl radical scavenging activity of hydrolysates; (**C**) ABTS radical scavenging activity of hydrolysates; (**D**) superoxide anion scavenging activity of hydrolysates; (**E**) reducing capacity of hydrolysates; and (**F**) metal-chelating activity of hydrolysates. Purple diamonds represent ascorbic acid; black boxes represent neutrase; red circles represent papain; green inverted triangles represent flavourzyme; and blue triangles represent alcalase.

Examination of the DPPH radical scavenging activity is a commonly used technique for assessing the in vitro antioxidant activity of compounds. The technique is based on the principle that DPPH provides maximum absorbance at 517 nm. The antioxidant activity of the hydrolysate is expressed as a decrease in absorbance or a decrease in the pure color intensity of the sample [25]. As shown in Figure 3A, the DPPH radical scavenging capacity of all samples was concentration-dependent and exhibited stronger scavenging ability with increasing concentration. The flavourzyme hydrolysates showed significantly higher scavenging activity than the other three proteases. At a concentration of 8 mg/mL, the scavenging activity of flavourzyme hydrolysates was comparable to that of ascorbic acid. Moreover, nearly all samples treated with flavourzyme or its combination exhibited relatively high DPPH radical scavenging activity [9].

Hydroxyl radicals are highly reactive free radicals found in biological tissues that can cause physiological disorders by reacting with proteins, DNA, and lipids. These radicals play an essential role in lipid peroxidation and hydrophilic oxidation [26]. Figure 3B shows that the hydroxyl radical scavenging activity increased significantly (*p* < 0.05) with increasing the sample concentration from 0.2 to 0.6 mg/mL and tended to level off at 0.6–1.0 mg/mL. The free radical scavenging activity of alcalase and papain hydrolysates at a concentration of 0.8 mg/mL was found to be similar to that of ascorbic acid. The strong hydroxyl radical scavenging activity can be attributed to the high content of hydrophobic amino acids [26]. It has been shown that the presence of a benzene ring group in aromatic amino acids acts as an oxidative chain breaker through the hydrogen-atom transfer (HAT) mechanism. In a study conducted by Kai Wang et al. [27], it was revealed that alcalase and papain hydrolysates contained higher levels of aromatic amino acids, which resulted in an increased hydroxyl radical scavenging capacity of these two hydrolysates.

The decolorization of the radical ABTS cation in the green solution (ABTS•^+^) is used to confirm the radical activity of the hydrolysates [28]. As depicted in Figure 3C, the ABTS radical scavenging activity of the CFHs was found to be concentration-dependent, with alcalase showing significantly higher (*p* < 0.05) activity than the other three proteases. This could be attributed to the synergistic effect of the peptides in the hydrolysate on the ABTS radical scavenging activity. The ABTS radical scavenging activity of the alcalase hydrolysate at a concentration of 1 mg/mL was found to be comparable to that of ascorbic acid. A study by Zohreh Karami et al. [13] revealed that the ABTS radical scavenging activity of hydrolysates from adzuki bean (*Vigna angularis*) and mung bean (*Vigna radiata*) protein concentrates was significantly higher (*p* < 0.05) using alcalase in comparison to using flavourzyme hydrolysates.

According to Figure 3D, the CFHs exhibited a lower superoxide anion scavenging activity (*p* < 0.05) compared to the ascorbic acid. No significant difference (*p* > 0.05) in scavenging activity between flavourzyme and alcalase was observed, but both showed higher activity than neutrase and papain. It is possible that CFHs contain inactive polypeptides, resulting in an overall lower clearance activity [21].

The potential antioxidant capacity of protein hydrolysates is often measured by their reduction capacity [29]. Figure 3E demonstrates that the reduction capacity of CFHs was concentration-dependent. The radical scavenging activity of the flavourzyme hydrolysate was significantly higher (*p* < 0.05) than the activity of the other three proteases. At 30 mg/mL, the activity of the flavourzyme hydrolysate was similar to that of the ascorbic acid. The reduction capacity was influenced by the concentration and type of proteases. This might be due to the fact that flavourzyme increased the number of free amino acids, thereby increasing the number of protons and electrons exposed and available for redox reactions [25].

As shown in Figure 3F, the metal-chelating activity of the CFHs increased as the concentration increased (*p* < 0.05). The hydrolysates of alcalase, papain, and neutrase all exhibited significantly higher chelating activity than that of the ascorbic acid, especially alcalase hydrolysates, showing the highest activity. Additionally, the flavourzyme hydrolysate demonstrated higher chelating activity than the ascorbic acid at concentrations above 0.8 mg/mL. Our findings were in agreement with those of Liu et al. [22] and Zohreh Karami et al. [13], who found that alcalase was more effective than flavourzyme, papain, and neutrase in producing metal-chelating peptides from adzuki bean (*Vigna angularis*) and mung bean (*Vigna radiata*) proteins. Alcalase is a highly effective protease with a wide range of specificity, capable of cleaving polypeptide bonds and releasing additional carboxyl and amino groups on branched chains, leading to the liberation of more acidic and basic amino acids, with a particular emphasis on Phe, Tyr, and Lys. The end result is an increase in the negative charge, which in turn facilitates the binding of Fe^2+^ radicals [13,22,30]. 

### 2.5. Effect of Different Proteases on the Pancreatic Lipase Inhibitory Activity of Hydrolysates

**Figure 4:**
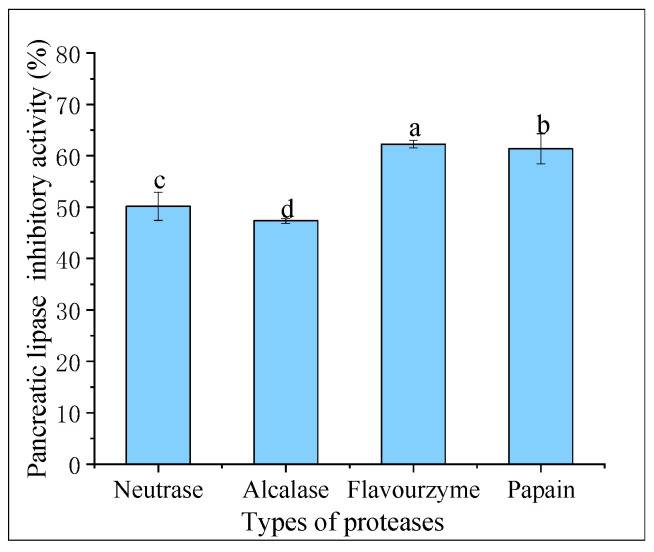
Pancreatic lipase inhibitory activity of hydrolysates. The lowercase letters indicate significant differences (*p* < 0.05) in the pancreatic lipase inhibitory activity of CFHs obtained by different proteases’ hydrolysis.

Pancreatic lipase is the primary enzyme responsible for breaking down triacylglycerols during fat digestion and absorption in the intestine. To limit intestinal fat absorption, pancreatic lipase inhibitors have been developed and shown to be effective in controlling hyperlipidemias, making them promising drugs for weight loss. Figure 4 displays the pancreatic lipase inhibitory activity of hydrolysates created from different proteases at 20 mg/mL. Flavourzyme hydrolysates had greater pancreatic lipase inhibitory activity (62.27% ± 0.73%) compared to those of papain (61.36% ± 2.93%), neutrase (50.18% ± 2.74%), and alcalase (47.34% ± 0.46%) hydrolysates. In a study conducted by Priti Mudgil et al. [31], it was found that the hydrolysates obtained through papain had superior pancreatic lipase inhibitory activity compared to that of those obtained through alcalase. Another recent study suggested that the pancreatic lipase inhibitory activity of CFHs was weaker than camel casein hydrolysates, which could be attributed to the longer enzymatic digestion time of CFHs [32].

### 2.6. Bioactivity Stability of CFHs after Simulated Gastrointestinal Digestion In Vitro

In recent times, in vitro digestion models have proven to be a valuable tool for comprehending the structural and chemical transformations that occur during simulated gastrointestinal conditions. These models have been widely employed to assess the digestibility, bioavailability, and physicochemical properties of peptides [33]. During digestion, the biological activity of hydrolysates can either be activated or inactivated. One of the crucial determinants of the bioactivity of peptides in vitro is their resistance to gastrointestinal digestion [15]. The study investigated the changes in the stability of antioxidant activity and pancreatic lipase inhibitory activity in CFHs obtained from different proteases during simulated gastrointestinal digestion in vitro.

#### 2.6.1. Antioxidant Activity

**Figure 5:**
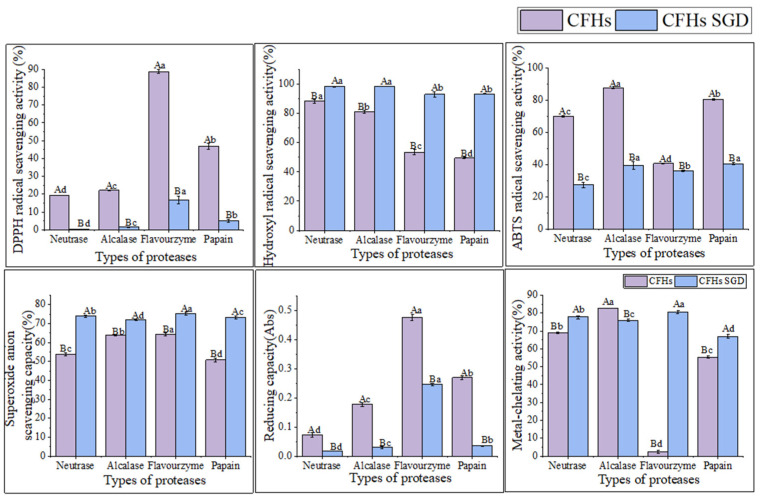
The antioxidant activity stability of CFHs before and after simulated gastrointestinal digestion in vitro. The uppercase letters represent significant differences (*p* < 0.05) in the activity of CFHs before and after simulated gastrointestinal digestion in vitro, while the lowercase letters indicate significant differences (*p* < 0.05) in the activity of CFHs obtained by different proteases’ hydrolysis.

According to the findings presented in Figure 5, the antioxidant activity of the CFHs persisted after simulated gastrointestinal digestion in vitro. The hydroxyl radical scavenging activity and superoxide anion scavenging activity were observed to be significantly higher (*p* < 0.05), while the DPPH radical scavenging activity, ABTS radical scavenging activity, and reduction capacity were significantly lower (*p* < 0.05). The results could be attributed to a decrease in the quantity of amino acids possessing antioxidant activity in CFHs during simulated gastrointestinal digestion in vitro [33]. The antioxidant activity of peptides is related to their structural characteristics such as hydrophobicity, sequence, and amino acid composition [33]. After simulated gastrointestinal digestion in vitro, the metal-chelating activity of hydrolysates obtained by each protease increased significantly (*p* < 0.05), except for that of the alcalase hydrolysates. The finding was consistent with a study conducted by Zhang et al. [15]. As shown in Figure 5, the flavourzyme CFHs exhibited higher gastrointestinal digestive stability than the other three proteases, indicating that the stability of the hydrolysates during gastrointestinal digestion was influenced by the protease used [15,33].

#### 2.6.2. Pancreatic Lipase Inhibitory Activity

**Figure 6:**
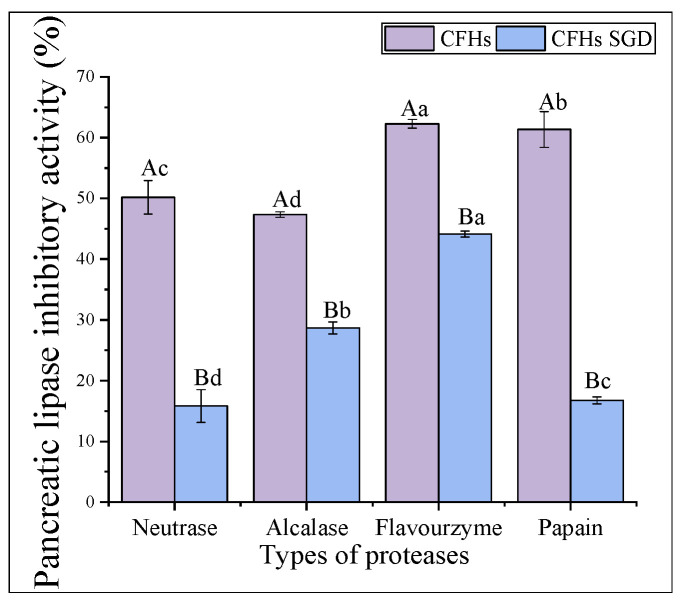
The pancreatic lipase inhibitory activity stability of CFHs. The uppercase letters represent significant differences (*p* < 0.05) in the activity of CFHs before and after simulated gastrointestinal digestion in vitro, while the lowercase letters indicate significant differences (*p* < 0.05) in the activity of CFHs obtained by different proteases’ hydrolysis.

As shown in Figure 6, the pancreatic lipase inhibitory activity of the hydrolysates obtained by all proteases decreased significantly (*p* < 0.05) after simulated gastrointestinal digestion in vitro. The highest inhibitory activity was observed in the flavourzyme after digestion (44.12% ± 0.49%). The results of the antioxidant activity stability were consistent with the above findings. The interaction of the peptide with phospholipase is inhibited by lipase [34]. Peptides that can bind more binding sites are dominated by hydrophobic amino acids, such as folic acid and proline [34]. The decrease in activity after simulated gastrointestinal digestion in vitro might be attributed to a reduction in the hydrophobic amino acid content of the individual hydrolysates after digestion.

## 3. Materials and Methods

### 3.1. Materials and Reagents

Fresh *Cucumaria frondosa* intestines and ovum were purchased from Haizhongbao seafood trading center (Yantai, China). Neutrase (50,000 U/g), alcalase (200,000 U/g), flavourzyme (15,000 U/g), and papain (100,000 U/g) were of food grade and were purchased from Solarbio Biotechnology Co. Ltd. (Beijing, China). All other reagents and chemicals used in this study were of analytical grade and were purchased from Sinopharm Chemical Reagent Co. Ltd. (Shanghai, China).

### 3.2. Preparation of Hydrolysates

As shown in Table 1, CFHs were prepared by hydrolyzing *Cucumaria frondosa* intestines and ovum with proteases at optimum temperature and mild agitation at 100 rpm in an orbital shaker incubator. Once the hydrolysis was complete, the mixture was heated in a boiling water bath for 15 min to stop the hydrolysis. After cooling the mixture in ice water, it was centrifuged at 10,000× *g* for 15 min at 4 °C, and the resulting supernatants were lyophilized to obtain the final CFH product.

### 3.3. Determination of DH

The DH was determined using a modified ninhydrin colorimetric method [35]. A sample was taken in a test tube and 1 mL of ninhydrin solution was added. The mixture was sealed with plastic wrap and heated in a boiling water bath for 15 min. After boiling, the tube was rapidly cooled in cold water and 5 mL of 40% ethanol solution was added. The solution was shaken well until it faded to brownish-red and left at room temperature for 10 min. The solution was zeroed with distilled water and the absorbance was measured at 570 nm.

DH (%) = h/h_tot_ × 100 (1)

where h represents the number of millimoles of peptide bonds cleaved per gram of sample (mmol), and h_tot_ represents the number of millimoles of peptide bonds per gram of sample (mmol). For this experiment, a value of 7.99 mmol/g was used based on the amino acid composition of the sample.

### 3.4. Solubility

The solubility of the proteins in the hydrolysates was determined according to the method described by Vásquez [36] with slight modifications. The hydrolysates were dissolved in distilled water at a concentration of 1% *w*/*v*, and the pH of the mixture was adjusted to 2.0, 4.0, 6.0, 8.0, and 10.0 using either 1 mol/L NaOH or 1 mol/L HCl. After centrifugation at 4000 rpm for 20 min, the soluble fraction (supernatant) was collected, and the protein content was determined using the Lowry and Randall [37] method. The percentage of solubility was calculated according to the following equation.

Solubility (%) = (Protein content of the supernatant)/(Protein content of the sample) × 100(2)

### 3.5. Structural Characteristics of CFHs

#### 3.5.1. Surface Hydrophobicity

The surface hydrophobicity of the samples was determined using the method developed by Zhang et al. [38]. We used 1-anilino-8-naphthalenesulfonate (ANS), a hydrophobic probe. Each sample was diluted with 0.1 M phosphate buffer (pH 7.0) to a concentration of 1 mg/mL and mixed with 20 μL of 8 mM ANS solution and 2 mL of ACH hydrolysate. The fluorescence intensity of each sample was measured at an excitation wavelength of 375 nm and a scanning (SHIMADZU, Tohoku, Japan) wavelength range of 400–650 nm.

#### 3.5.2. UV-Vis Spectroscopy

A UV-Vis spectrum (UV-Vis Spectroscopy, METASH, Shanghai, China) was obtained by dissolving 15 mg of the CFHs in 10 mL of ultrapure water to acquire a concentration of 1.5 mg/mL and scanning in the range of 200–600 nm.

#### 3.5.3. FTIR

Two mg samples of enzymatic hydrolysates were compressed with potassium bromide. The infrared spectrometer (Perkin Elmer, Waltham, MA, USA) was utilized for a full-spectrum scan with a resolution of 4 cm^−1^, ranging from 4000 to 400 cm^−1^. The Spectrum 10.4.1 software version was employed for infrared spectrum mapping and data collection.

#### 3.5.4. Intrinsic Fluorescence Spectroscopy

The fluorescence spectra of protein hydrolysates were measured through fluorescence spectrophotometry (SHIMADZU, Tohoku, Japan), following the method outlined by Du et al. [24]. The excitation wavelength was set at 290 nm, and the emission spectrum range was set from 330–550 nm with a gap width of 5 nm. Prior to measurement, the sample was diluted to a concentration of 0.2 mg/mL in a phosphate buffer with a pH of 7.0.

### 3.6. Antioxidant Activity of CFHs

#### 3.6.1. DPPH Radical Scavenging Activity

The DPPH radical scavenging activity of protein hydrolysate was determined as described by Du Mx [39] with slight modifications. The samples were prepared at different concentrations (4.0, 5.0, 6.0, 7.0, and 8.0 mg/mL) and mixed with DPPH (0.2 mM) in equal proportions. The mixture was allowed to stand in the dark for 30 min, and the absorbance was monitored at 517 nm using a microplate reader (Thermo Scientific, Pleasanton, CA, USA). Anhydrous ethanol was used as the blank control, and VC was used as the positive control. The DPPH radical scavenging activity was calculated as follows:

DPPH radical scavenging activity (%) = [1 − (A_1_ − A_2_)/A_0_] × 100 (3)

where A_0_ represents the absorbance value of anhydrous ethanol instead of sample, A_1_ represents the absorbance value of the sample group, and A_2_ represents the absorbance value of anhydrous ethanol instead of DPPH.

#### 3.6.2. Hydroxyl Radical Scavenging Activity

The hydroxyl radical scavenging activity was determined using a modified method based on Zhou et al. [40]. In short, a mixture of 1.0 mL FeSO_4_ (2 mM), 1.0 mL salicylic acid-ethanol solution (6 mM), and 1.0 mL sample (0.2, 0.4, 0.6, 0.8, and 1.0 mg/mL) was prepared. To the mixture, 1.0 mL H_2_O_2_ (6 mM) was added and incubated at 37 °C for 30 min. The absorbance of the mixture was immediately measured at 510 nm using a microplate reader (Thermo Scientific, USA). The hydroxyl radical scavenging activity was calculated using the following formula:

Hydroxyl radical scavenging activity (%) = [1 − (A_2_ − A_1_)/A_0_)] × 100 (4)

where A_0_ represents the absorbance value of distilled water instead of the sample, A_1_ represents the absorbance value of distilled water instead of the hydrogen peroxide solution, and A_2_ represents the absorbance value of the sample mixed with ferrous sulfate, hydrogen peroxide, and salicylic acid ethanol solution.

#### 3.6.3. Superoxide Anion Scavenging Activity

The superoxide anion scavenging activity was measured with reference to the method developed by Xie et al. [41]. Samples of 0.5 mL each (1.8, 2.0, 2.2, 2.4, and 2.6 mg/mL) were mixed with 5.0 mL of Tris-HCl buffer solution (50 mM, pH 8.2) and incubated in a water bath at 25 °C for 20 min, followed by the addition of 0.5 mL of 3 mM pyrogallol solution pre-heated to 25 °C. The absorbance values were measured at 325 nm every 30 s for 5 min. Ascorbic acid was used as a positive control. The superoxide anion scavenging activity was calculated using the following equation:

Superoxide anion scavenging activity (%) = (1 − A_1_/A_0_) × 100 (5)

where A_1_ represents the absorbance value of the sample group, and A_0_ represents the absorbance value of the blank group.

#### 3.6.4. Reduction Capacity

To determine the reduction capacity, the method of Liu et al. [42] was used with some modifications. A total of 1 mL of each CFH at different concentrations (10.0, 15.0, 20.0, 25.0, and 30.0 mg/mL) was mixed with 2.5 mL of 1% potassium ferricyanide solution and 2.5 mL of 0.2 M phosphate buffer (pH 6.6). The resulting mixture was placed in a 50 °C water bath for 20 min and cooled to room temperature, and 2.5 mL of 10% TCA was added. After centrifugation at 3000 rpm for 10 min, 2.5 mL of the supernatant was transferred into a test tube, then 2.5 mL of distilled water and 0.5 mL of 0.1% FeCl_3_ were added. The absorbance values of the mixture were measured at 700 nm using an enzyme marker (Thermo Scientific, CA, USA). An increase in the absorbance value indicated an increase in the reduction capacity.

#### 3.6.5. Metal-Chelating Activity

The Fe^2+^ chelating activity was determined as follows [21]: 100 μL of the sample at concentrations of 4.0, 6.0, 8.0, 10.0, and 12.0 mg/mL, along with 135 μL of distilled water and 5 μL of 2 mM FeCl_2_, were mixed together. After 3 min, 10 μL of 5 mM iron reagent was added to the mixture. The mixture was then shaken at 25 °C and allowed to stand for 10 min. Finally, the absorbance was measured at 562 nm using a microplate reader (Thermo Scientific, USA). The absorbance of the blank was also determined using distilled water. The metal-chelating activity was determined as follows:

Metal-chelating activity (%) = (1 − A_1_/A_0_) × 100 (6)

where A_1_ represents the absorbance value of the sample group and A_0_ represents the absorbance value of the blank group.

#### 3.6.6. ABTS Radical Scavenging Activity

The ABTS radical scavenging ability of the CFHs was determined using the method described by Liu et al. [42]. A solution of ABTS (7.0 mmol/L) and potassium persulfate (2.45 mmol/L) was mixed at a 1:1 ratio and left to react in the dark for 12 h. The resulting solution was diluted with ethanol to an absorbance of approximately 0.70 ± 0.05 at 734 nm and stored in a dark environment. Samples with varying concentrations (0.2, 0.4, 0.6, 0.8, and 1 mg/mL) were mixed with ABTS solution at a 1:8 ratio and incubated for 10 min at 37 °C. The absorbance was measured at A_734_ and the ABTS radical scavenging ability was calculated using the following formula:

ABTS radical scavenging activity (%) =(1 − A_1_/A_0_) × 100 (7)

where A_0_ represents the absorbance value of the blank group and A_1_ represents the absorbance value of the sample group.

### 3.7. Pancreatic Lipase Inhibitory Activity

According to the method by Fisayo et al. [43], 50 μL of different CFH samples, along with 25 μL of 5 mM p-nitrophenyl butyrate and 55 μL of sodium phosphate buffer (100 mM, pH 7.4, containing 100 mM NaCl), were mixed together and added to a 96-well microplate. The mixture was preincubated at 37 °C for 5 min. The reaction was initiated by the addition of 20 μL of 50 mg/mL pancreatic lipase, and the final volume was adjusted to 150 μL with assay buffer. The reaction plate was then incubated at 37 °C for 30 min, and the absorbance of released p-nitrophenyl produced for each test sample was recorded at 405 nm using a microplate reader (Thermo Scientific, USA). The pancreatic lipase inhibitory activity was determined using the following equation:

Pancreatic lipase inhibitory activity (%) = [A_1_ − (A_2_ − A_3_)]/A_1_ × 100 (8)

where A_1_ represents the absorbance value without adding a sample, A_2_ represents the absorbance value after adding the sample, and A_3_ represents the absorbance value after adding the sample but without adding the substrate and enzyme solution.

### 3.8. Simulated Gastrointestinal Digestion In Vitro of Hydrolysates

The hydrolysates underwent digestion in vitro according to the method of Minekus et al. [44]. In brief, a 5 mL of sample was combined with 7.5 mL of simulated gastric fluid containing 1.6 mL of pepsin (2000 U/mL). The mixture was stirred for 2 h at 37 °C at a speed of 200 rpm. The gastric phase was then interrupted by adjusting the pH to 7.0 with 1 mol/L NaOH. Next, 5 mL of trypsin solution (100 U/mL) and 11 mL of simulated intestinal fluid were added to the digested sample. The mixture was then stirred for another 2 h at 37 °C at a speed of 200 rpm. After completion, the final mixture was immediately placed in ice water for precooling for 10 min, followed by refrigeration at −40 °C for 10 min to halt the trypsin reaction. The resulting hydrolysates from the gastric and gastrointestinal phases were collected, frozen, and freeze-dried for subsequent analysis.

### 3.9. Statistical Analysis

The experimental data were tested in triplicate, and the results are presented as the average ± standard deviation (*n* = 3). The ANOVA analysis was performed using SPSS 13.0 software (SPSS Inc., Chicago, IL, USA).

## 4. Conclusions

In this study, we investigated the structural characteristics, antioxidant activities, pancreatic lipase inhibitory activity, and stability of hydrolysates produced using four different proteases. The selection of protease types is crucial for the production of bioactive hydrolysates as it can significantly impact their biological activity. We found that the alcalase hydrolysate exhibited the highest surface hydrophobicity, which can be attributed to the exposure of numerous hydrophobic groups. As a result, the alcalase hydrolysates demonstrated superior antioxidant activity, including hydroxyl radical scavenging activity, ABTS radical scavenging activity, and metal-chelating activity compared to the other hydrolysates. Meanwhile, the flavourzyme hydrolysate had the highest DH and exhibited the highest DPPH radical scavenging activity, reduction capacity, and pancreatic lipase inhibitory activity. Papain, on the other hand, exhibited a high hydroxyl radical scavenging activity and demonstrated the highest solubility across different pH levels. Additionally, the hydrolysates of flavourzyme displayed excellent antioxidant stability and showed pancreatic lipase inhibitory activity during simulated gastrointestinal digestion in vitro. Based on these findings, the hydrolysates of *Cucumaria frondosa* intestines and ovum prepared using flavourzyme were identified as the optimal choice for the production of functional foods with biological activity. These results suggest that CFHs have the potential to be utilized as antioxidants and for their hypolipidemic activity in functional foods, dietary supplements, and nutraceuticals.

## Figures and Tables

**Table 1:** Hydrolysis conditions of different proteases.

Proteases	Temperature (°C)	pH	Solid–Liquid Ratio (*w/v*)	Proteases Addition (U/g Protein)	Time (h)
Neutrase	50	7	1:20	6000	7
Alcalase	60	10.5	1:20	7000	7
Flavourzyme	35	5.5	1:15	10,000	9
Papain	60	7.5	1:8	7000	7

## Data Availability

The data that support the findings of this study are available from the corresponding author, upon reasonable request.
